# A case series describing the risk of periodontal disease in Marfan syndrome patients harboring a possible aortic aneurysm or dissection

**DOI:** 10.1186/s12903-022-02361-5

**Published:** 2022-08-09

**Authors:** Kouta Umezawa, Takako Kajiwara, Kyoko Ishii, Tatsuya Hasegawa, Shigeto Suzuki, Masato Nakano, Mayu Sawaguchi, Venkata Suresh Venkataiah, Yoshio Yahata, Koki Ito, Yoshikatsu Saiki, Masahiro Saito

**Affiliations:** 1 Division of Restorative Dentistry, Department of Ecological Dentistry, Tohoku University Graduate School of Dentistry, Sendai, Miyagi Japan; 2 Division of Cardiovascular Surgery, Tohoku University Graduate School of Medicine, Sendai, Miyagi Japan

**Keywords:** Marfan syndrome, Periodontal conditions, Periodontal disease, Aortic aneurysm and dissection, Oral hygiene

## Abstract

**Background:**

Marfan syndrome (MFS) is a systemic disorder of connective tissues caused by insufficient elastic fiber formation that leads to structural weakness and results in various tissue disorders, including cardiovascular and periodontal disease. Notably however, the risk of periodontal disease in MFS patients affected by an aortic aneurysm or dissection has not yet been clarified.

**Methods:**

We investigated the periodontal condition in the following three groups: MFS patients diagnosed with an aortic aneurysm or dissection with a planned aortic surgery (MFS surgery), MFS patients who had already undergone aortic surgery (MFS post-surgery) and healthy control patients (Healthy). The periodontal condition of all of these patients was evaluated at their first visit, reassessed again at two-month after the first visit, and evaluated again at a six-month follow-up after the reassessment.

**Results:**

A total of 14 participants, 3 MFS surgery patients, 4 MFS post-surgery patients and 7 healthy control volunteers were examined. Saliva examinations revealed no significant differences between any of the groups at the first visit, reassessment, or follow-up. Interestingly, the MFS surgery cases showed a higher BOP and PISA at the first visit and follow-up compared with the other groups. In contrast, the MFS surgery patients showed an improvement in their LVDd and EF values, both markers of cardiac function, at the reassessment and follow-up compared with the first visit.

**Conclusions:**

MFS associated with an aortic aneurysm or dissection leads to a higher risk of periodontal disease, indicating the need for more frequent oral hygiene maintenance in these patients. In addition, MFS patients who undergo frequent professional cleaning of their teeth show a lower onset of cardiovascular disease, suggesting that professional oral hygiene in these cases contributes to a healthier condition.

**Supplementary Information:**

The online version contains supplementary material available at 10.1186/s12903-022-02361-5.

## Introduction

Marfan syndrome (MFS) is an autosomal dominant disorder of connective tissue that affects approximately 1 in 5000 people. It is caused by missense mutations of Fibrillin-1 (FBN-1), a component of the extracellular microfibrils, leading to cardiovascular complications including aortic root dilatation, dissection, and rupture which are the leading causes of morbidity and mortality [1, 2]. Blood vessel prosthesis implantation surgery, such as aortic replacement therapy, to replace a disorganized artery or cardiac valve, and pharmacotherapeutics such as β blockers, have been used to prevent the rate of aortic root dilatation in MFS patients [3]. Although these treatments have been shown to produce quality of life improvements, the prognosis from the perspective of cardiac health remains poor in MFS patients, many of whom require an aortic replacement [4]. Other known complications of MFS including ocular lens dislocation, emphysema, joint hypermobility, and periodontal disease, are also exacerbated by progression of cardiac adverse events [5]. Among these other complications, the onset of periodontal disease, which causes soft tissue destruction and progressive bone destruction leading to tooth mobility and subsequent loss, leads to a greater risk of contracting bacterial infective endocarditis. The prevention and treatment of periodontal disease are therefore critical for the management of MFS patients [6].

Periodontal disease is caused by a bacterial infection that activates the innate immune response via Toll-like receptors, resulting in the up-regulation of innate immunity cytokines such as Tumor Necrosis Factor-α(TNF-α), Interleukin-1(IL-1), and Interleukin-6(IL-6) to ultimately cause progressive tissue destruction [7]. MFS has been shown to increase the susceptibility to severe periodontal disease, in association with periodontal ligament dysfunction, due to microfibril insufficiency, suggesting that FBN-1 microfibril formation plays a central role in periodontal ligament formation [8, 9]. Notably in this regard, the elastic fibers of the periodontal ligament, known as oxytalan fibers, primarily consist of FBN-1 microfibrils and do not contain significant amounts of elastin. Hence, periodontal ligaments were found to be more susceptible than other connective tissues to breakdown in an MFS mouse model [10]. In our previous study, periodontal disease in FBN-1-deficient mice (FBN-1c1039G/+mice), an MFS model mice which developed aortic aneurysm and dissection, interfered with wound healing of diseased periodontal tissue due to a continuous expression of inflammatory markers such as TNF-alpha and matrix metalloproteinase-9 (MMP-9) [11]. These findings suggested that high susceptibility to periodontal disease was present along with cardiac complications in patients with MFS.

In contrast to the findings of these aforementioned studies, another clinical study reported that MFS cases did not show high susceptibility to periodontal disease but showed more inflammation of periodontal tissue [6]. However, this discrepancy seems to be related to the status of cardiac complications, such as the presence of an aortic aneurysm or dissections, or a valvulopathy. MFS patients with any signs of these cardiac disorders seems also have exacerbated connective tissue destruction, suggesting that these phenomena increase the susceptibility to periodontal disease. In contrast, MFS patients who managed to control such cardiac-related symptoms have been reported to show a low risk of periodontal disease due to suppressed connective tissue destruction [12]. To verify this hypothesis, a clinical study that compared periodontal disease in MFS patients with or without the presence of cardiac issues was required. In our present study therefore, we investigated the periodontal disease status and its treatment effects in MFS patients who also had symptoms of an aortic aneurysm or dissection and had a planned aortic surgery, in a further group of MFS patients in whom cardiac complications had already been improved by surgical treatment and pharmacotherapeutics, and in a healthy patient control group. We evaluated whether the cardiac health status in MFS affects the progression of periodontal disease, and if this could be alleviated by initial treatments and oral cleaning guidance.

## Materials and methods

### Patients

The present study was approved by the Ethical Committee of Tohoku University in accordance with the Declaration of Helsinki (No. 2018-3-025) and was designed as a case control investigation. The case subjects were MFS patients for whom an aortic surgery had been scheduled (MFS surgery), or who were followed-up after aortic surgery (MFS post-surgery). Patients with no systemic disease including cardiovascular disease, were employed as a healthy volunteer (healthy control group). All the study subjects were ensured not having active caries lesions prior to start this study. MFS patients who were diagnosed in accordance with the established clinical criteria for this disorder [13] were recruited from the Division of Cardiovascular Surgery of Tohoku University Hospital. Any patients with a history of mental illness were excluded from further analysis due to the possible impacts of this on the study findings. Detailed instructions were given to the enrolled subjects, including a brochure explaining the study design, and written informed consent was obtained in all cases. This study was conducted between November 2018 and August 2020. Some information on the physical condition of the MFS patients and the results of cardiac examinations of these cases was collected from Division of Cardiovascular Surgery at Tohoku University Hospital.

### Periodontal examinations

All of the study participants underwent a comprehensive periodontal examination, including oral photography (9 sheets method) and a plaque control record. Probing pocket depth (PPD) and bleeding on probing (BOP) tests were conducted at six sites per tooth (disto-buccal, buccal, mesio-buccal, mesio-oral, oral, disto-oral) to calculate the periodontal inflamed surface area (PISA), a useful index of the inflammation status for clinical and epidemiological assessments [14]. We also investigated mean PPD, a number of furcation involvement and tooth mobility to evaluate the clinical periodontal status.

**Fig. 1:**
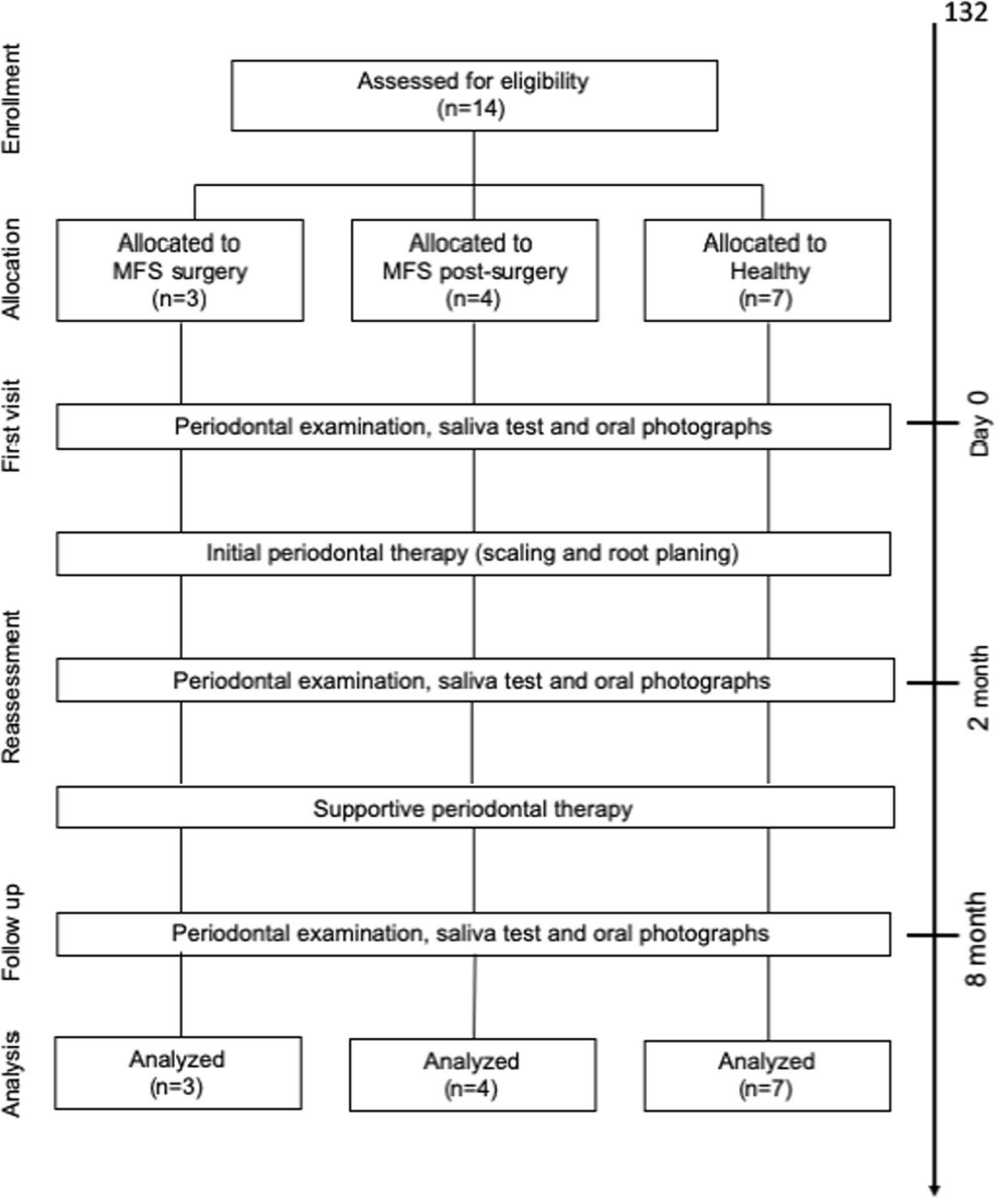
Study design

All measurements, including periodontal examinations, saliva tests and oral photographs, were carried out by an experienced dental hygienist at the first visit. Within two months after this first visit, initial periodontal treatments including scaling and root planning were performed. The effects of the initial periodontal treatments were evaluated by periodontal examination after initial periodontal treatment as a reassessment. All of the study subjects visited clinics every two months for supportive periodontal therapy (SPT) after reassessment, their periodontal condition was evaluated after six months as a follow-up, and these treatments were continued as maintenance therapy (Fig. 1). Adequate oral hygiene instructions were given to all of the study participants during the initial periodontal treatment and SPT.

All of the study subjects underwent an initial periodontal treatment, including scaling and root planing after their first visit for two months. Reassessments were carried out at two months after the first visit. Supportive periodontal therapy was performed every two months after the reassessment. Follow-up examinations were carried out at six months after the reassessment. All of the study participants underwent periodontal examinations via saliva tests and oral photographs at the first visit, reassessment and follow-up.

### Cardiac examinations

Cardiac function was evaluated by assessing the left ventricular end-diastolic diameter (LVDd) and ejection fraction (EF) in the surgery and post-surgery MFS patients.

### Statistical analysis

The recorded data were documented and analyzed using the data procession program JMP Pro 15 (SAS Institute Inc, Cary, NC). Each individual subject counted as a statistical unit in all tests. The mean values and range were calculated for all parameters. The Tukey–Kramer method was applied to determine significant differences between the groups. For all tests, a P-value of ≤ 0.05 was considered statistically significant.

## Results

### Study participants

**Fig. 2:**
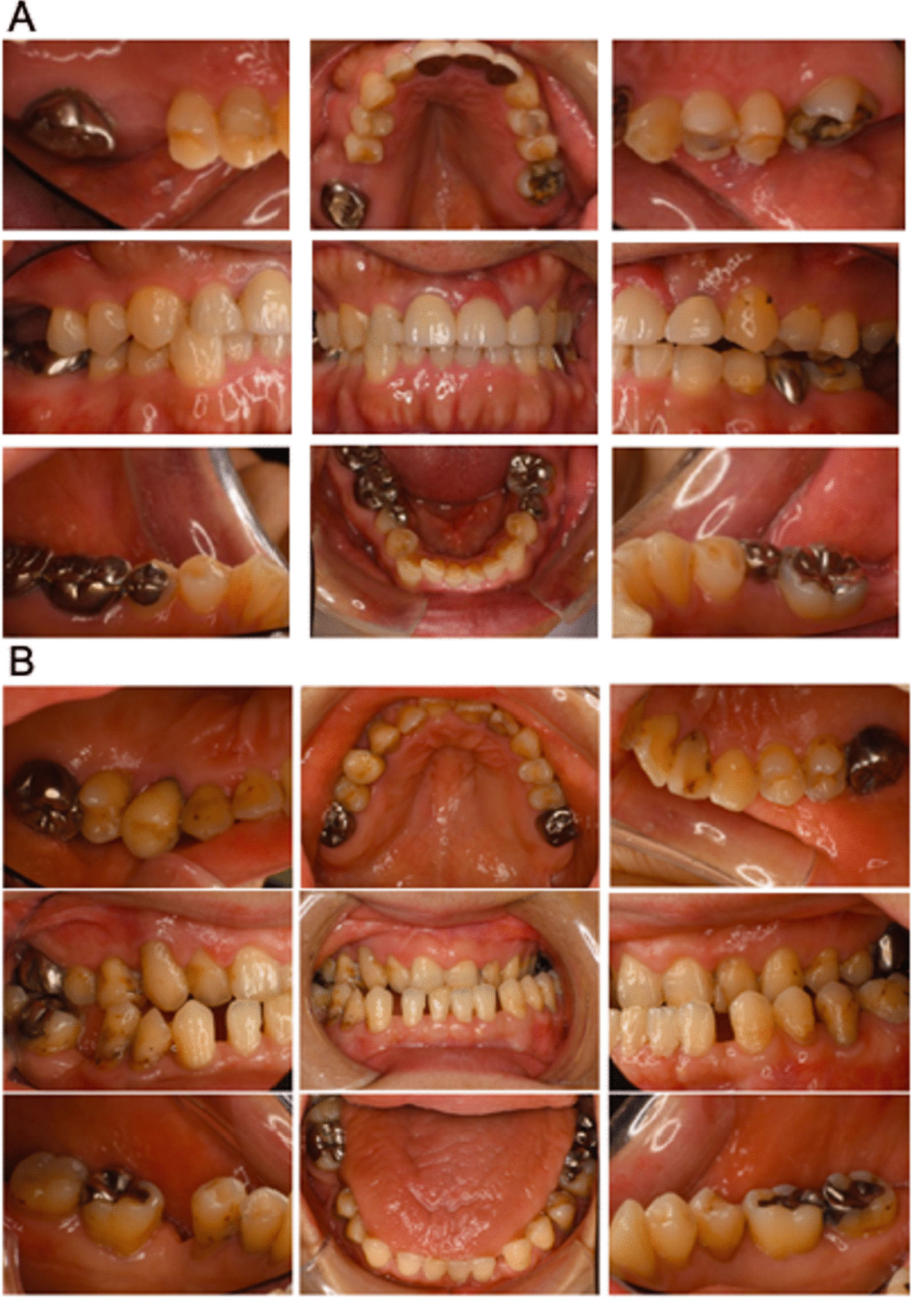
Oral photographs of the Marfan syndrome patients. **a** Oral photographs taken at the first visit in the MFS patients with a planned aortic surgery. **b** Oral photographs taken at the first visit in the MFS post-surgery group

A total of 14 participants 3 MFS surgery patients (1 female and 2 males, mean age: 65.3 years); 4 MFS post-surgery patients (2 females and 2 males, mean age: 43.8 years), and 7 healthy volunteers (6 females and 1 male, mean age: 46.1 years), were included in this study (Table 1). Oral photographs indicated that both the MFS surgery group (Fig. 2a) and MFS post-surgery group (Fig. 2b) subjects had a high palate and malocclusion. Examination of All of the study participants were started at November 2018 for their periodontal condition at the first visit, followed by a reassessment, and received SPT up to March 2020. Visits to the clinic were not possible for the 5 months prior to that date due to the COVID 19 pandemic.

**Table 1:** Patient characteristics

	MFS surgery	MFS post-surgery	Healthy
Subject number	3	4	7
Male/female	2 (male), 1 (female)	2 (male), 2 (female)	1 (male), 6 (female)
Age	65.3 ± 10.6	43.8 ± 1.5	46.1 ± 17.0

### Periodontal examinations

To investigate the risk of periodontal disease, periodontal examinations including PPD, number of mobile teeth, number of teeth with furcation involvement, plaque control record, BOP, and PISA tests were also conducted at the first visit, reassessment, and 6-month follow-up. Mean PPD, number of mobile teeth, number of teeth with furcation involvement and plaque control record did not show significant difference in each group at first visit, reassessment and 6-month follow-up (Table 2). In all groups, the plaque control record decreased from the first visit to the reassessment and increased slightly from the reassessment to the follow-up period.

**Table 2:** Results of mean PPD, number of mobile teeth, teeth with furcation involvement and plaque control record

	MFS surgery	MFS post-surgery	Healthy
*Mean PPD (mm)*			
First visit	2.6 ± 0.4	2.4 ± 0.2	2.3 ± 0.2
Reassessment	2.5 ± 0.3	2.3 ± 0.2	2.1 ± 0.1
Follow up	2.7 ± 0.3	2.3 ± 0.3	2.2 ± 0.2
*Number of mobile teeth*			
First visit	0.3 ± 0.6	1 ± 1.2	0
Reassessment	0.3 ± 0.6	1 ± 1.2	0
Follow up	0.3 ± 0.6	1 ± 1.2	0
*Number of teeth with furcation involvement*			
First visit	0.3 ± 0.6	0	0
Reassessment	0.3 ± 0.6	0	0
Follow up	0.3 ± 0.6	0	0
*Plaque control record*			
First visit	22.3 ± 6.9	33.3 ± 30.8	19.7 ± 10.7
Reassessment	19.7 ± 8.7	18.3 ± 7.4	10.4 ± 8.6
Follow up	25.7 ± 9.8	25.7 ± 14.5	16.7 ± 23.0

**Fig. 3:**
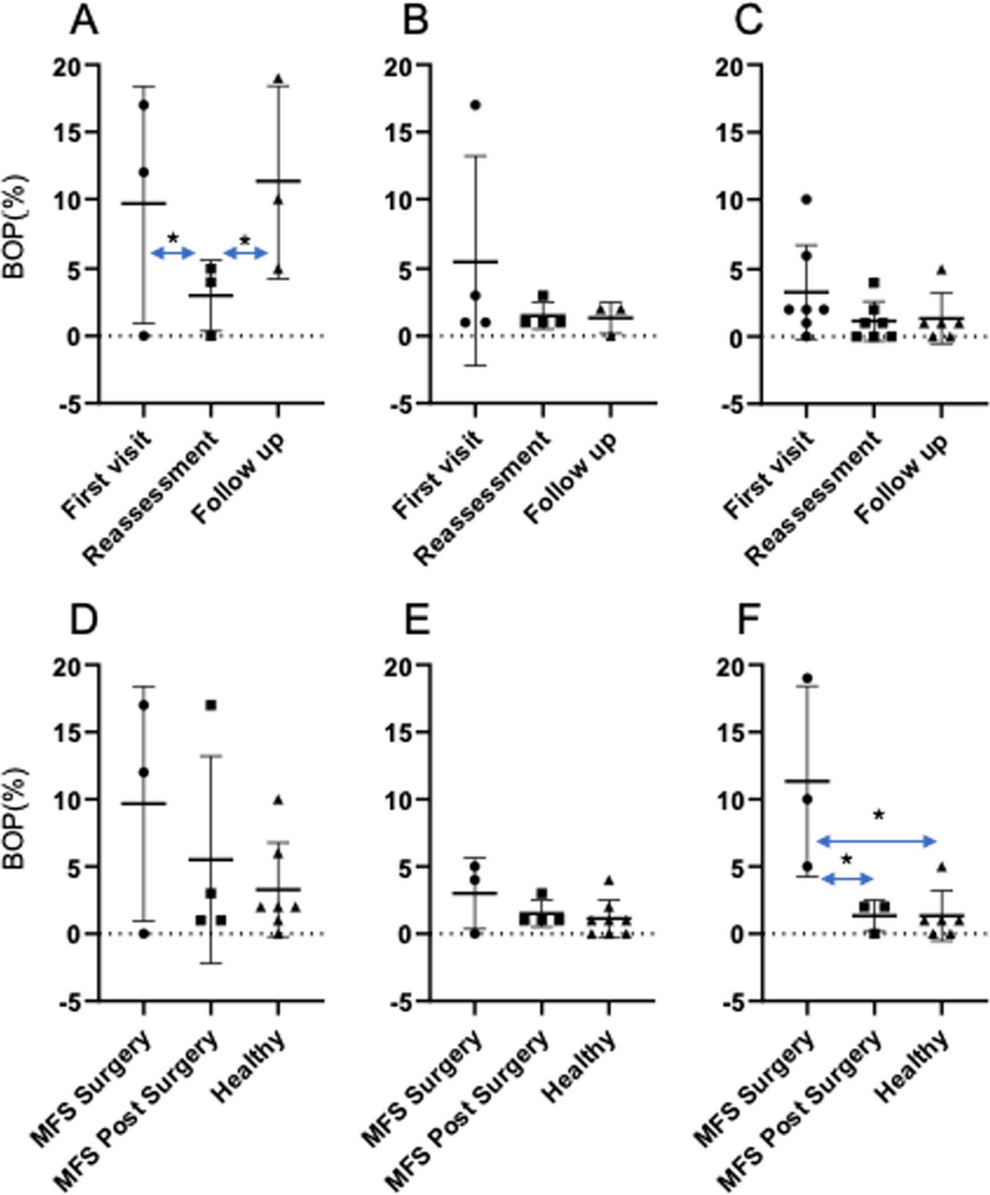
Comparison of the BOP test results between the study groups at each examination point. **a** BOP outcomes in the MFS surgery group between examinations. **b** BOP outcomes in the MFS post-surgery group between examinations. **c** BOP outcomes in the Healthy group between examinations. **d** Comparison of the BOP outcomes at the first visit between the groups. **e** Comparison of the BOP outcomes at the reassessment examinations between the groups. **f** Comparison of the BOP outcomes at the follow-up examinations between groups. *P < 0.05

**Fig. 4:**
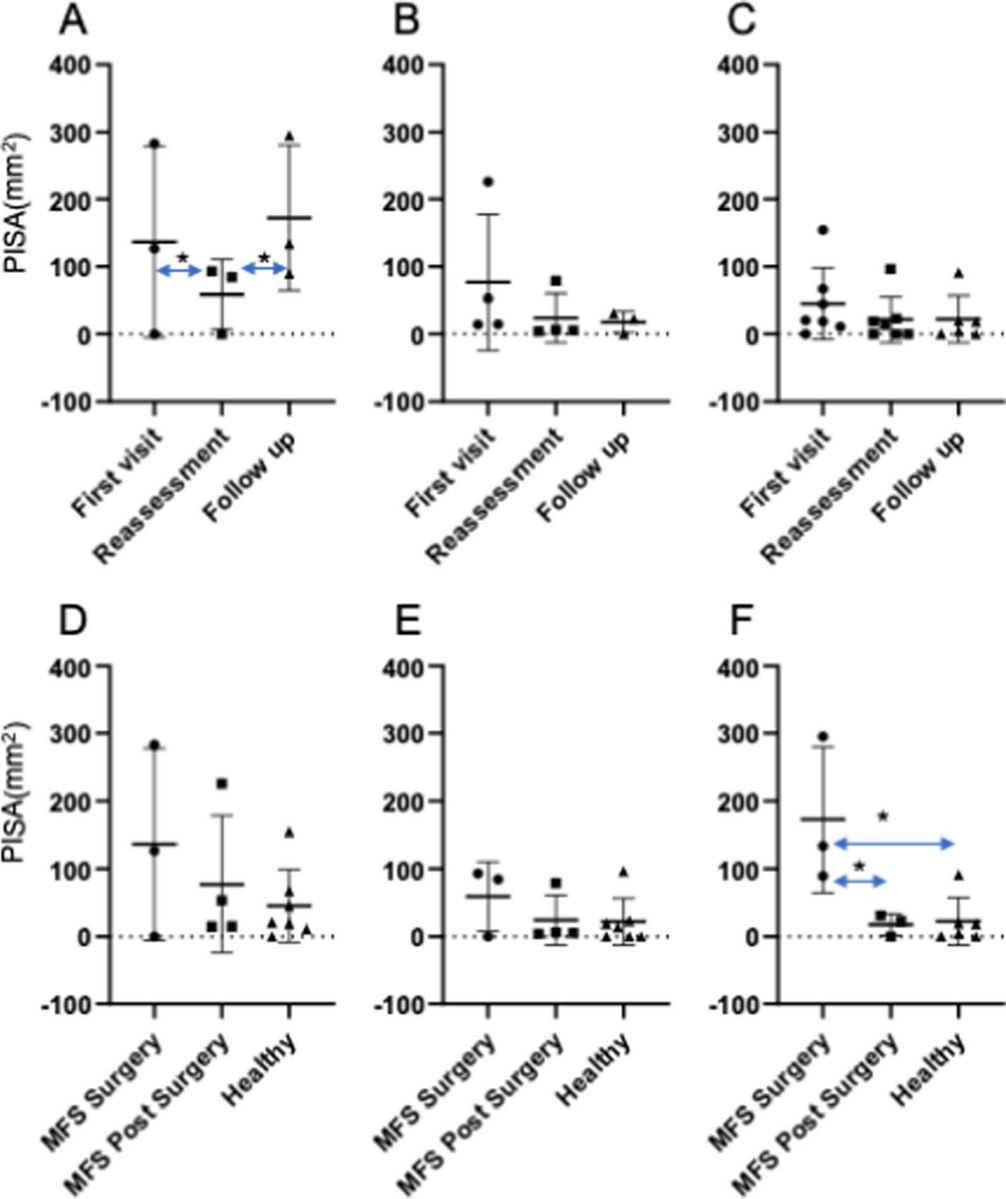
Comparison of the PISA test results between the study groups at each examination point. **a** PISA outcomes in the MFS surgery group between examinations. **b** PISA outcomes in the MFS post-surgery group between examinations. **c** PISA outcomes in the Healthy group between examinations. **d** Comparison of the PISA outcomes at the first visit between the groups. **e** Comparison of the PISA outcomes at the reassessment examinations between the groups. **f** Comparison of the PISA outcomes at the follow-up examinations between groups. *P < 0.05

The MFS surgery patients showed a higher BOP and PISA than the subjects in the MFS post-surgery or Healthy groups (Figs. 3d, 4d) and these were found to be decreased significantly from the first visit to the reassessment after the initial treatment (Figs. 3a, 4a). After the reassessment however, the MFS surgery patients were unable to visit a clinic for SPT due to the COVID 19 pandemic. Both the BOP and PISA scores were increased significantly from the reassessment to the follow-up in the MFS surgery group (Figs. 3a, 4a). By contrast, there were no significant differences found in the BOP and PISA data obtained at the first visit, reassessment or follow-up between the MFS post-surgery, and Healthy groups (Figs. 3e, f, 4e, f). In addition, decreases in both the BOP and PISA were apparent from the first visit to the reassessment and follow-up in MFS post-surgery, and Healthy groups even though visits could not be made the clinic after the reassessments (Figs. 3b, c, 4b, c). Each participant in the MFS-surgery performed follow-up examination at 340 days (MFS surgery1), 372 days (MFS surgery2), 369 days (MFS surgery3) after cardiac surgery.

**Fig. 5:**
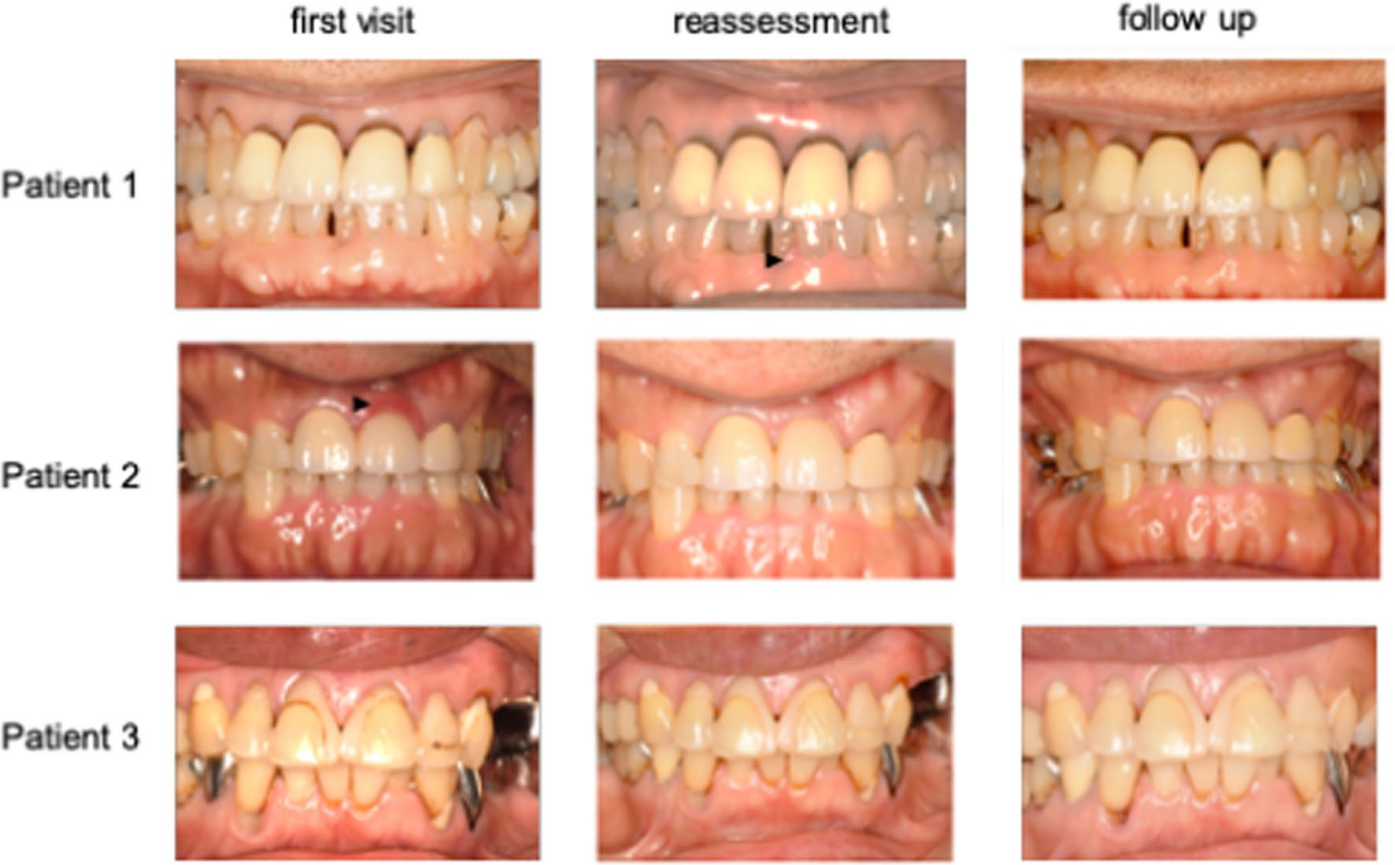
Oral photographs of the MFS surgery subjects

Changes in the oral condition of the MFS surgery group subjects are indicated in Fig. 5. Patient 1 showed no characteristic features of periodontal disease at the first visit but displayed gingival swelling at the mandibular anterior teeth at the reassessment. Notably however, the gingival condition in this subject had almost recovered to normal at the follow-up stage. Gingival swelling and redness around the maxillary anterior teeth were evident in MFS surgery Patient 2 at the first visit, but these were no longer detectable at the reassessment or follow-up. Patient 3 in this group exhibited no characteristic periodontitis features at any of the three examination stages.

Oral photographs indicating changes over time for each patient in the MFS surgery group. Swelling and redness of the gingiva were evident in Patient 1 at the reassessment examination (arrowhead) and in Patient 2 at the first visit (arrowhead).

### Cardiac function

We next evaluated the cardiac function status during periodontal treatment at the first visit, reassessment, and follow-up period in the MFS surgery and post-surgery groups using the established LVDd and EF indicators (Table 3). All of the MFS surgery patients showed improvement in the LVDd and EF values at the reassessment and follow-up period compared to the first visit. The MFS post-surgery group cases showed improvement in cardiac function during the whole period of periodontal treatment.

**Table 3:** Status of cardiac function during periodontal treatment

		LVDd (mm)*	EF (%)*
*MFS surgery*			
Patient1	First visit	54	57
	Reassessment	51	67
	Follow up	49	73
Patient2	First visit	58	66
	Reassessment	56	67
	Follow up	56	65
Patient3	First visit	52	66
	Reassessment	42	66
	Follow up	-	-
*MFS post-surgery*			
Patient4	First visit	48	65
	Reassessment	46	71
	Follow up	58	66
Patient5	First visit	55	62
	Reassessment	52	57
	Follow up	54	65
Patient6	First visit	55	67
	Reassessment	54	68
	Follow up	53	67
Patient7	First visit	46	69
	Reassessment	44	61
	Follow up	44	66

*Standard value of Left Ventricular end-Diastolic diameter (LVDd) and Ejection Fraction (EF) was 41–52 mm or 59–71% respectively

## Discussion

We here provide the first report that has compared periodontal disease activity between MFS surgery, MFS post-surgery, and healthy control subjects during a long-term observation period to further investigate whether MFS increases the risk of periodontal disease. Although it was difficult to recruit suitable subjects due to the rare prevalence of MFS in this study, our results suggested that the periodontal disease status worsened in the MFS surgery patients after a prolonged gap in follow-up maintenance due to the COVID-19 pandemic. From these findings, we proposed that professional intervention decreases the onset of periodontal disease activity in MFS patients. However, more frequent maintenance was required in MFS patients who received surgery to treat aortic aneurysm and dissection. In addition, the cardiovascular function was unaffected during treatment for oral issues in the MFS surgery and post-surgery groups, suggesting that professional intervention may contribute to a more stable health condition in these patients. Major cardiovascular manifestation of MFS can include a thoracic aortic aneurysm, mitral valve prolapse (MVP) and cardiac dysfunction [15]. The reported histopathological findings are elastic lamellae fragmentation and disorganized aortic tissue architecture due to an insufficient microfibril assembly [16]. The major component of the elastic fiber in the periodontal ligament is fibrillin-1 and this underlies why periodontal tissue is one of the most affected connective tissue in MFS [17]. Indeed, Shiga et al. provided evidence of obvious and severe PDL disorganization in the mgR/mgR mouse model of MFS, in which aortic aneurysms or dissection develop, along with severe periodontal tissue destruction [9]. Previous clinical studies have revealed that a more severe type of periodontitis can manifest in MFS patients, according to a case report from one patient using gingival inflammation analysis [8]. Two somewhat controversial clinical studies were subsequently published, one of which reported that MFS patients do not have a higher prevalence of periodontitis, while the other reported a high prevalence [6, 7]. Staufenbiel et al. reported that MFS patients show a slight tendency towards more periodontal inflammation but not a higher prevalence or more frequent onset of severe forms of periodontitis than healthy controls, suggesting that mutation of fibrillin-1 in MFS does not cause a higher susceptibility to this disease [6]. In contrast, Suzuki et al. indicated in their study that MFS patients diagnosed with cardiovascular disease, including aortic aneurismal and cardiac valvular disorders, develop severe periodontitis more frequently than non-MFS cases with a cardiovascular disorder [12]. In that study also, the serum titer levels for antibodies against periodontal pathogens such as *Prevotella intermedia* were significantly lower in MFS patients with cardiovascular disease compared to the non-MFS cardiovascular disease cases [12]. In addition, Venza et al. also reported that a young age in MFS subjects was associated with a high amount of plaque and gingival inflammation [18]. In our present study, we observed that the MFS surgery subjects has a slight tendency towards increased periodontitis, but that this was not a significant difference. Interestingly, these MFS surgery cases showed a higher susceptibility to periodontitis at a one-year follow-up compared to the MFS post-surgery and Healthy groups. Previously reported findings have suggested that MFS patients suffer from a high degree of inflammation because of crowded teeth, which makes it challenging to maintain good oral hygiene [6]. Another recent study has reported that an aortic aneurysm related to MFS was associated with tooth pain [19]. Taken together with previous findings and our current data, MFS cases prior to a planned aortic surgery seem to have a lower activity host defense system than their post-surgery counterparts. Hence, careful and frequent oral hygiene maintenance is required in MFS patients prior to cardiovascular surgery. The cumulative evidence to date indicates that MFS patients who have symptoms of an aortic aneurysm or dissection and have undergone aortic surgery show a higher degree of periodontal disease activity compared with MFS cases who are being maintained on a pharmacotherapeutic regimen due to the release of inflammatory cytokines in the gingival tissue which will promote a higher susceptibility to periodontal disease. Our current data also suggest that time will be required to lower the onset of disease activity of MFS after surgery because our MFS surgery cases showed a higher risk of periodontal disease at follow-up after surgery.

The management of MFS patients who develop aortic aneurysms includes medical therapy and prophylactic surgery to reduce the risk of an aortic dissection [20, 21]. However, dysfunctional fibrillin increases the bioavailability of transforming growth factor-beta (TGF-β), leading to the activation of proinflammatory transcription factors and an increased expression of matrix metalloproteinases and inflammatory cytokines in the aorta. All of these processes can promote further elastic fiber degradation, which increases the risk of vascular complications. Losartan, an angiotensin receptor blocker (ARB), has been used for the prevention of aneurysms in mouse models to stabilize the aortic root in pediatric patients with MFS and also to ameliorate aggressive aortic disease by blocking TGF-β signaling [23]. Beta blockers are prescribed to normotensive individuals with MFS and blood pressure control is an important part of the management of MFS. Even after treatment with these therapeutics, an aortic aneurysm can occur after aortic replacement, suggesting that gradual aortic lamina degradation progresses continuously in these patients. Similar to this exacerbated aortic lamina degradation, our present data have suggested that the risk of complications including periodontal disease increases in MFS patients who have yet to undergo aortic surgery. Thus, the risk of periodontal disease in MFS patients appears to affect the possible onset of an aortic aneurism or dissection. This may explain why periodontitis was exacerbated in our current MFS surgery patients by the prolonged follow-up period due to the coronavirus pandemic. To further elucidate these questions, further investigations are necessary to analyze whether inflammatory markers, including metalloproteases or inflammatory cytokines, are released into the oral environment of MFS patients with a planned aortic surgery.

## Conclusion

Professional oral hygiene prevents the risk of periodontal disease in MFS patients diagnosed with cardiovascular disease and regular examinations are required in these cases where an aortic surgery is planned to reduce the risk of periodontal disease. In addition, none of the MFS patients analyzed in this study showed a progression of their cardiovascular disease, suggesting that a professional approach to oral hygiene contributes to maintaining a healthy condition in these cases.

## Supplementary Information

**Additional file 1**.** Supplementary Table 1**. Results from buffer capacity,* S.mutans*, and* Lactobacillus* testing.

## Data Availability

The datasets generated and/or analysed during the current study are available in the figshare repository, https://doi.org/10.6084/m9.figshare.19228110.v1.
