# Supplementary material for: A case series describing the risk of periodontal disease in Marfan syndrome patients harboring a possible aortic aneurysm or dissection
Source: BMC Oral Health. 2022 Aug 9;22:336. doi: 10.1186/s12903-022-02361-5 (PMC9360735; doi:10.1186/s12903-022-02361-5)
Supplement: Supplementary file 1 — Additional file 1. Supplementary Table 1. Results from buffer capacity, S.mutans, and Lactobacillus testing. [file 12903_2022_2361_MOESM1_ESM.docx]

A Case series describing the risk of periodontal disease in Marfan syndrome patients

harboring a possible aortic aneurysm or dissection

Kouta Umezawa^1^, Takako Kajiwara^1^, Kyoko Ishii^1^, Tatsuya Hasegawa^1^, Shigeto Suzuki^1^, Masato Nakano^1^, Mayu Sawaguchi^1^, Venkata Suresh Venkataiah^1^, Yoshio Yahata^1^, Koki Ito^2^, Yoshikatsu Saiki^2^, Masahiro Saito^1*^

^1^Division of Restorative Dentistry, Department of Ecological Dentistry, Tohoku University Graduate School of Dentistry, Sendai, Miyagi, Japan

^2^Division of Cardiovascular Surgery, Tohoku University Graduate School of Medicine, Sendai, Miyagi, Japan

Supplementary information

| Buffer capacity score | 0 | pH≧6.0 |
| --- | --- | --- |
|  | 1 | pH4.5〜5.5 |
|  | 2 | pH≦4.0 |
| S.Mutans score | 0 | ≦10,000CFU/ml |
|  | 1 | 10,000〜100,000CFU/ml |
|  | 2 | 100,000〜1,000,000CFU/ml |
|  | 3 | ≧1,000,000CFU/ml |
| Lactobaccillus score | 0 | 1,000CFU/ml |
|  | 1 | 10,000CFU/ml |
|  | 2 | 100,000CFU/ml |
|  | 3 | 1,000,000CFU/ml |

Supplementary Table 1 Results from buffer capacity, *S.mutans*, and *Lactobacillus* testing
